# Proximity-based vocal networks reveal social relationships in the Southern white rhinoceros

**DOI:** 10.1038/s41598-020-72052-0

**Published:** 2020-09-15

**Authors:** Julia Jenikejew, Brenda Chaignon, Sabrina Linn, Marina Scheumann

**Affiliations:** 1 Institute of Zoology, University of Veterinary Medicine Hannover, Bünteweg 17, 30559 Hannover, Germany; 2 Université Bourgogne Franche-Comté, 21078 Dijon, France; 3 Zoo Frankfurt, 60316 Frankfurt, Germany

**Keywords:** Behavioural ecology, Animal behaviour

## Abstract

Vocal communication networks can be linked to social behaviour, allowing a deeper understanding of social relationships among individuals. For this purpose, the description of vocal dyads is fundamental. In group-living species, this identification is based on behavioural indicators which require a high level of reactivity during social interactions. In the present study, we alternatively established a proximity-based approach to investigate whether sex-specific differences in vocal communication reflect social behaviour in a species with rather loose social associations and low levels of reactivity: the Southern white rhinoceros (*Ceratotherium simum simum*). We performed audio- and video recordings of 30 captive animals from seven groups. Vocal networks for the four most common call types were constructed by considering conspecifics at close distance (≤ 1 body length) to the sender as potential receivers. The analysis of the resulting unidirectional structures showed that not only the sex of the sender but also the sex of the potential receiver, the quality of social interactions (affiliative or agonistic) as well as association strength predict the intensity of vocal interactions between group members. Thus, a proximity-based approach can be used to construct vocal networks providing information about the social relationships of conspecifics—even in species with loose social associations where behavioural indicators are limited.

## Introduction

Communication is a key factor for animals with regard to reproduction and survival as it enables group living by mediating social behaviour, foraging as well as fight-or-flight behaviour^1,2^. Vocal communication, comprising conspicuous and far-ranging signals, is of special interest as it serves many crucial functions such as territorial defence, inter-specific recognition, group synchronisation and associating over distance^1,3,4^. Characterising vocal communication contributes to a more profound understanding of a group’s social structure as well as directionality and types of social interactions even in species that are spatially scattered^4^.

The most basic constellation in vocal communication is the dyadic relationship between a sender and a receiver^5^. In species communicating within audible range, senders can be tracked by auditory localisation of the sound source, entailing a rather obvious identification. In contrast, identification of the receiver is not as apparent as there are often several individuals available within the signalling range^4^. In order to resolve this issue, most studies on animal vocal communication assess the potentially intended receiver by means of behavioural indicators such as vocal exchange (e.g.^6–13^), bodily reactions (e.g. gazing at the receiver or moving towards the sender^14,15^) or the situational context (e.g. affiliative^16–18^ or agonistic interactions^19,20^). Vocal communication networks use the dyadic sender-receiver units to illustrate complex relationships between several signallers and receivers within a group^2,21^. Thereby, signalling links are visualised in interrelated arrangements based on call occurrence, call directionality or call rate^4^. These arrangements can be used to investigate structural patterns such as sex-specific associations and characteristics^22^.

Sexual dimorphism has long been of great interest in mammalian species^23^. Sexual selection as well as the adaptation to sex-specific niches are presumed to be the underlying driving forces that manifest in the form of genetic, morphological, physiological and behavioural variations^24^. Over the last decades, particular attention has been paid to sexual dimorphisms in vocal behaviour. Several studies showed that male and female senders can differ in overall vocal activity (e.g.^25–28^) as well as their vocal repertoire (e.g.^29–33^). Sex differences can even be found in shared call types regarding call rate (e.g.^14,30,34^), acoustic structure (e.g.^28,29,33,35–39^) and the behavioural context the calls are uttered in (e.g.^31^). These sex-specific differences in vocal behaviour have been found among a variety of mammalian taxa (e.g. rodents^28^, bats^38^, felines^34^, ungulates^32^, elephants^39^ and primates^25^). In addition to being affected by the sex of the sender, vocal behaviour has also been proven to be affected by the sex and identity of the receiver. Thus, studies could show an effect of audience in mammalian vocal communication structures, implying that the identity of a receiver, such as its sex or kinship, also influences the sender’s calling behaviour (e.g.^3,4,28,40–42^).

As vocal interactions can be linked to the quality of interactions between sender and receiver, vocal communication structures can be used in order to reflect social relationships (e.g.^7,43^). For example, a positive correlation between grooming and contact-calling networks was found in a study conducted in a free-ranging ring-tailed lemur group^8^, highlighting that vocal interactions indicate strong affiliative bonds between conspecifics. However, it is not clear to which extent sex-specific differences in vocal behaviour are related to the quality of social interactions between sexes and how social systems might affect this link, as sex-specific differences in vocalisation occur in solitary (e.g.^33,34^), pair- (e.g.^27,30,44^) as well as group-living species (e.g.^14,26,29,35^). The majority of mammalian species involved in vocal communication studies live in large groups and are therefore eminently social and known to be highly responsive to conspecifics, which provides a thoroughly suitable basis for the detection of behavioural indicators of vocal interactions (e.g.,^7,8^). Snijders and Naguib^4^ argued that vocal structures of group-living species can fundamentally differ from species with comparatively loose spatial associations and low levels of interactions (e.g., territorial species). However, insights into vocalisation structures in these kinds of species remain unexplored for the most part.

The Southern white rhinoceros (*Ceratotherium simum simum,* SWR) is described as a ‘semi-social’ megaherbivore inhabiting the savannah grasslands of the Southern African continent^45,46^. Adult males are solitary, only associating with females when they are approaching oestrus, as well as territorial, occasionally accepting subordinate satellite bulls^46–50^. In contrast, adult females form temporarily stable groups with other females and subadults for up to several months or even years^46,47,51^. In addition to their very pronounced olfactory sense, SWR have a distinct vocal repertoire with ten to eleven different call types^46,52^, of which the most common call types in adult animals are Hiss, Grunt, Pant and Snort (in previous publications Hiss has been termed Threat, but in order to be consistent all call types are labelled onomatopoeically from here on). Whereas Hiss and Grunt are uttered exclusively during agonistic interactions, Pants seem to function as contact as well as mating calls, while Snorts are mainly uttered during resting and feeding^46,52,53^. Despite the well depicted vocalisations, determining communication structures in SWR groups still poses a challenge with regard to the identification of vocal interaction partners, as the majority of obvious reactions to approaching conspecifics or vocalisations are only observed during infrequent high-intensity encounters such as fighting or mating^50^. Thus, in the present study we used a proximity-based approach to investigate vocal communication structures.

Spatial measurements are frequently used by researchers in order to characterise social networks, as they are often more feasible than collecting actual interaction data^4^. Thereby, spatial proximity can be classified in various ways, e.g. by defining a set distance measurement, identifying nearest neighbours, grouping individuals using the ‘chain rule’ or assigning individuals to the same location^54,55^. We transferred these approaches to the vocal communication network analyses and defined the potential receiver based on its distance to the sender during vocalisation. In doing so, we expected the sender to display higher call rates when potentially intended receivers were at close distance.

Accordingly, the aim of the present study was to test whether a proximity-based approach can be used to investigate sex-specific differences in the vocal behaviour of the SWR. Using parameters of distinct vocal communication structures, we investigated whether call rates and call rate distributions in vocal networks differed between male and female senders. In order to understand the underlying causes for differences in call rates, we further analysed the effects of three potential factors predicting the varying call rates: Sex of the potential receiver, quality of the interactions (affiliative or agonistic) and the association strength between sender and potential receiver.

## Methods

### Study sites and animals

Acoustic and behavioural recordings were performed on 30 Southern white rhinoceroses (*Ceratotherium simum simum*) that were kept in seven groups at different zoological institutions: Zoo Osnabrück (N = 4), Zoo Augsburg (N = 4), Zoopark Erfurt (N = 3), Serengeti-Park Hodenhagen (N = 9), ZOOM Gelsenkirchen (N = 3), Zoo Schwerin (N = 3) and Zoo Münster (N = 4). All animals were observed in their outside enclosures with the whole group. Each group consisted of one adult bull, two to four adult cows and in two zoos there were additional juvenile and subadult animals (Zoo Münster: one calf, Serengeti-Park Hodenhagen: three calves and one subadult female; Table 1).

**Table 1:** Information on study subjects and data collection. M = male, F = female, age in years, total observation time in hours.

ID	Sex	Zoo	Year of birth	Age during observation [year]	Total observation time [h]	Number of observation days
Floris	M	Osnabrück	1976	38	10	20
Lia	F	Osnabrück	2002	12	10	20
Marsita	F	Osnabrück	2005	9	10	20
Amalie	F	Osnabrück	2007	7	10	20
Bantu	M	Augsburg	2005	9	10	17
Baby	F	Augsburg	1971	43	10	15
Kibibi	F	Augsburg	2005	9	10	17
Chris	F	Augsburg	2005	9	10	18
Dino	M	Erfurt	1993	22	11	22
Numbi	F	Erfurt	1996	19	11	22
Temba	F	Erfurt	1997	18	11	22
Martin	M	Hodenhagen	1993	22	3	8
Molly	F	Hodenhagen	1972	42	5	16
Claudia	F	Hodenhagen	1998	17	5	17
Kianga	F	Hodenhagen	2004	11	5	17
Uzuri	F	Hodenhagen	2005	10	5	17
Lara	F	Hodenhagen	2011	4	5	16
Dinari	M	Hodenhagen	2013	2	4	17
Tatu	F	Hodenhagen	2013	2	5	16
Makena	F	Hodenhagen	2013	2	5	16
Lekuru	M	Gelsenkirchen	2004	11	10	20
Tamu	F	Gelsenkirchen	1992	23	10	18
Cera	F	Gelsenkirchen	1994	21	10	19
Kimba	M	Schwerin	2008	10	10	23
Karen	F	Schwerin	2003	15	10	23
Clara	F	Schwerin	2006	12	10	23
Harry	M	Münster	1990	28	10	22
Jane	F	Münster	1999	19	10	22
Vicky	F	Münster	1986	32	10	22
Amiri	M	Münster	2017	1	10	22

### Data collection

Video and audio recordings of the animals were taken simultaneously over an average period of 19 observation days at each zoo. All individuals were recorded using focal animal sampling^56^. Each focal individual was observed for ten minutes in a randomised order within groups, resulting in 20–40 min of observation time per individual distributed between 8 am and 6 pm. In total, 250 h of data were recorded and analysed: 40 h at Zoo Osnabrück (April 2014), 40 h at Zoo Augsburg (July/August 2014), 33 h at Zoopark Erfurt (April/May 2015), 37 h at Serengeti-Park Hodenhagen (April/May 2015), 30 h at ZOOM Gelsenkirchen (August/September 2015), 30 h at Zoo Schwerin (April/May 2018) and 40 h at Zoo Münster (July/August 2018).

Video recordings were made using a digital camcorder (Sony DCR-SR36E, Sony Corporation, Tokyo, Japan). Audio recordings were made using an omni-directional microphone (Sennheiser MKH 8020, Sennheiser electronic GmbH & Co. KG, Wedemark-Wennebostel, Germany; flat frequency response from 10–20,000 Hz ± 5db) that was equipped with a wind shield and a boom pole. The microphone was connected to a digital recording device (Sound Devices 702 T State Recorder, Sound Devices LLC, Reedsburg, USA; frequency response: 10–40,000 Hz; settings: 44.1 Hz sampling rate, 16 Bit, uncompressed .wav format).

### Vocal and behavioural analysis

Video recordings were synchronised with respective audio recordings and analysed using the *Observer XT* software (version 12, Noldus Information Technology, Netherlands^57^). The analysis was conducted by three different observers (CL: Erfurt, Gelsenkirchen; BC: Osnabrück, Hodenhagen, Schwerin; JJ: Augsburg, Münster). The Cohen’s Kappa coefficient was determined among the observers by comparing 15 pilot observations (total of 100 min). All *Ƙ* values were ≥ 0.95, indicating a high interrater reliability^58^.

**Figure 1:**
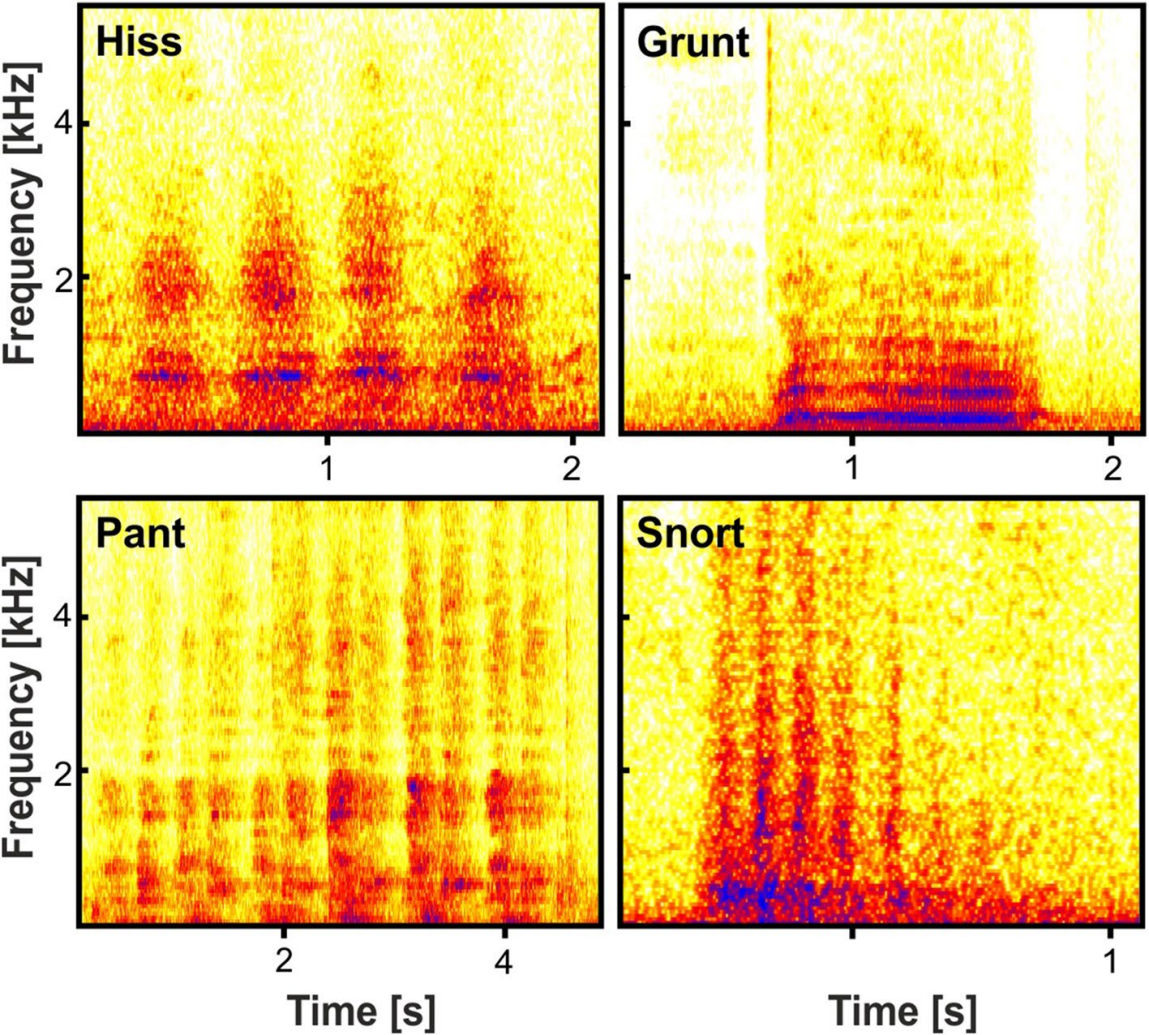
Spectrograms of Hiss, Grunt, Pant and Snort calls of adult Southern white rhinoceroses. Spectrograms were produced using *BatSound* (version 4.2, Pettersson Elektronik AB, Uppsala, Sweden; settings: FFT 512, Hanning window).

Vocalisations were detected by auditory identification and categorised according to the literature^46,52,53^. For each vocalisation, the respective call type and sender were noted. Vocalisations that due to ambient noises could not be reliably assigned a call type or a sender were excluded from the analysis. For a sufficient data basis, only call types that were uttered by at least 50% of adult senders were considered for further analysis. Consequently, the following four call types were included (Fig. 1): Hiss (N = 1,169), Grunt (N = 75), Pant (N = 83) and Snort (N = 1,027).

For each behaviour proximity measurements were coded, defining the distance of the focal animal to present group members. Due to practical as well as methodical reasons, adult body length (2.5–3 m^46^) was taken as a measuring unit. At one adult body length the distance could be reliably detected on video recordings, while allowing immediate social interactions between group members, therefore enabling the recording of behavioural context. Two distance categories were established: (1) close proximity (≤ 1 body length) and (2) distant proximity (> 1 body length). This differentiation ensured a reliable distinction that was not impaired by the distortion of the camera angle or by neighbouring individuals at great distances not being caught by the camera. For each focal animal the duration they spent in close proximity (≤ 1 body length) to each group member was noted. All group members in close proximity to the sender during vocalisations were considered as potential receivers.

For each focal animal the occurrence of affiliative, aggressive and defensive interactions (see ethogram in Table 2) and the respective interaction partners were noted. Affiliative interactions included social exploration of the interaction partner as well as socio-positive and sexual behaviour. Aggressive interactions were coded when the focal animal displaced, attacked, chased, pushed or clashed horns etc. with the interaction partner whereas defensive interactions were coded when the focal animal avoided or escaped from the interaction partner.

**Table 2:** Ethogram of affiliative and agonistic behaviour of captive Southern white rhinoceroses.

Interaction type	Behaviour	Description
Affiliative	Following	Focal animal moves after a conspecific, while changing the location
	Snout contact	Focal animal explores the body of another conspecific (except the snout) with its snout
	Social Flehming	Focal animal flehms, while scenting a defecating/ urinating conspecific close by
	Naso-nasal sniffing	Focal animal contacts the nasal region of another conspecific with its own snout
	Ano-genital sniffing	Focal animal contacts the ano-genital region of another conspecific with its snout
	Head placing	Focal animal lays its head on the back of another conspecific
	Body contact	Focal animal touches or brushes another conspecific with any part of its body (except snout) or rubs itself against a conspecific
	Presenting	Focal animal lifts up its tail while the bull is standing behind (only for cows)
	Mounting	Focal animal climbs with its forelegs on another conspecific
	Copulation	The animals mate: the bull inserts his penis into the cow
Aggressive	Displace	Focal animal incites a conspecific to change its position/location after approaching or agonistic interaction
	Nodding	Focal animal swings its head back and forth
	Lifting	Focal animal lifts another conspecific’s head or leg with its head/horns
	Staring	Focal animal stands horn to horn in front of another conspecific with an uplifted head
	Pushing	Focal animal presses any part of its body against another conspecific making it change the position/location
	Chasing	Focal animal *follows* another conspecific, which tries to keep the Focal animal at a distance, in a trotting manner
	Feigned attacking	Focal animal moves with a lowered head towards another conspecific and stops suddenly without causing body contact
	Attacking	Focal animal hits its horns against another conspecific
	Horn clashing	Escalated confrontation following *Attacking* involving both animals hitting their horns against each other
Defensive	Avoiding	Focal animal changes its position/location after being approached by a conspecific, agonistic interaction with it or agonistic vocalisation from it
	Escaping	Focal animal moves away from a conspecific in a trotting manner after an agonistic interaction

### Statistical analysis

All statistical tests were calculated in *RStudio* (version 3.5.5,^59^). The significance level was set at *p* ≤ 0.05, *p* < 0.1 was considered a statistical trend.

#### Communication networks and directionality

We constructed vocal communication networks for each call type in all groups based on the dyadic call rates for each dyad in the respective group. As the number of calls emitted to a potential receiver was dependent on the time the dyad spent in close proximity (≤ 1 body length) to each other, the daily dyadic call rate was calculated by dividing the number of calls the focal animal uttered to a potential receiver by the total duration the focal animal spent in close proximity (contact time) to the potential receiver on each observation day. Based on this, the dyadic call rate was calculated as a mean of the daily dyadic call rate.

Dyadic call rates were converted into directed adjacency matrices that were used to generate communication networks for each call type in all groups. Networks were drawn in *Gephi* (version 9.0.2,^60^). In the networks each individual was represented by one node and the ties between them indicated how the nodes related to one another. Nodes were set to a random position. The thickness of the ties represented the dyadic call rates, while the colour of the ties corresponded to the colour of the sender. In a network, a node indegree was defined as the total number of ties (group members) that was directed towards an individual, while the node outdegree was the total number of ties that originated from the respective individual^22,61,62^. Correspondingly, weight indegree was considered the sum of all the ties’ weights (sum of dyadic call rates) that were directed towards an individual, while the weight outdegree was the sum of all the ties’ weights that originated from the respective individual^61,62^.

Communication networks included all animals available in the groups. However, for further analysis we excluded juvenile individuals (≤ 2 years, N = 4), as we expected sex-specific differences to start developing strongest in individuals that were weaned and not depending on the mothers anymore.

To investigate communication directionality, a Pearson correlation between the node in- and outdegrees was performed for each call type using the ‘cor.test’ function. Non-significant correlations were categorised as asymmetrical directionality and significant correlations as symmetrical directionality.

#### Sex-specific sender differences

To assess sex-specific differences in general vocal activity, we calculated the overall call rate for each focal animal and for each call type by dividing the number of calls by the total observation time of the respective focal animal.

To investigate sex-specific differences in the proportion of incoming and outgoing call parameters, we calculated the ratio between node in- and outdegree (ION) and the ratio between weight in- and outdegree (IOW). ION was calculated by the number of node indegrees minus the number of node outdegrees divided by the sum of node in- and outdegrees. Correspondingly, IOW was calculated in the same manner by using the weights of the ties. Index values ranged between − 1 and + 1, with 0 indicating equal distribution of incoming and outgoing parameters. Accordingly, in individuals with negative index values, the outgoing degree was higher than the incoming degree, while in individuals with positive index values, the outgoing degree was lower than the incoming degree (Supplementary 1).

Sex-specific sender differences in overall call rates as well as in ION and IOW indices were determined by calculating linear mixed effects models (LMEs, ‘nlme’ package, ‘lme’ function,) using ‘sex of the sender’ as predictor variable while controlling for ‘zoo’ as random factor. Residuals were calculated for all LMEs performed in the study (‘resid’ function) and subsequently verified for normality as well as for homogeneity of variances.

#### Effects of dyad type, quality of social interaction and association strength

To determine the quality of social interactions, interaction rates for affiliative, aggressive and defensive social interactions were calculated for each focal animal-interaction partner dyad in a group. Thus, daily interaction rates were calculated by dividing the number of a) affiliative, b) aggressive or c) defensive interactions of the focal animal with an interaction partner by the total observation time of the focal animal on that day. Based on this, the interaction rate was calculated as the mean of the daily interaction rates.

To assess the association strength (AS) between dyad partners as a measurement for the level of social cohesion, the daily association index was calculated for each dyad by dividing the sum of the duration animal A spent in close proximity to animal B and animal B spent in close proximity to animal A by the sum of total observation times of both animals on that day. Based on this, AS was calculated as the mean of the daily association indices. AS values ranged from 0 to 1 with 1 indicating the strongest possible association, the dyad spending 100% of their observation time in close proximity to each other.

We further investigated whether dyadic call rates can be predicted by the sex composition of the dyad (dyad type: female-female (N = 40), female-male (N = 19), male–female (N = 19)), the quality of social interactions (affiliative, aggressive, defensive) or the association strength between both dyad partners. To determine this, LMEs were calculated for each call type using the dyadic call rate as response variable, two-way interactions between dyad type and social interaction type or between dyad type and association strength as predictor variables (dyad type*affiliative interaction rate, dyad type*aggressive interaction rate, dyad type*defensive interaction rate, dyad type*association strength), while controlling for “sender” and “zoo” as random factors. The best fitting model (final model) was determined via backward stepwise elimination procedure (‘car’ package, ‘Anova’ function). If the interaction term was not significant, only the main factors remained in the final model. To investigate significant effects of the main factor dyad type, a comparison across the three dyad types was conducted (‘lsmeans’ package, ‘lsmeans’ function, ‘Tukey’ adjustment for multiple comparisons). If an interaction term turned out to be significant, a break down analysis was performed by splitting the dataset according to dyad type. In the result section, only final models were reported along with slope β values indicating the coherence between call rate and the quality of social interaction or association strength.

### Ethical statement

The article contains only observational data of zoo animals during their daily routine. No animal was taken out of its usual environment or physically manipulated by the authors. The authors received permission to record the animals’ data on the ground of the respective zoo.

## Results

### Communication networks and directionality

**Figure 2:**
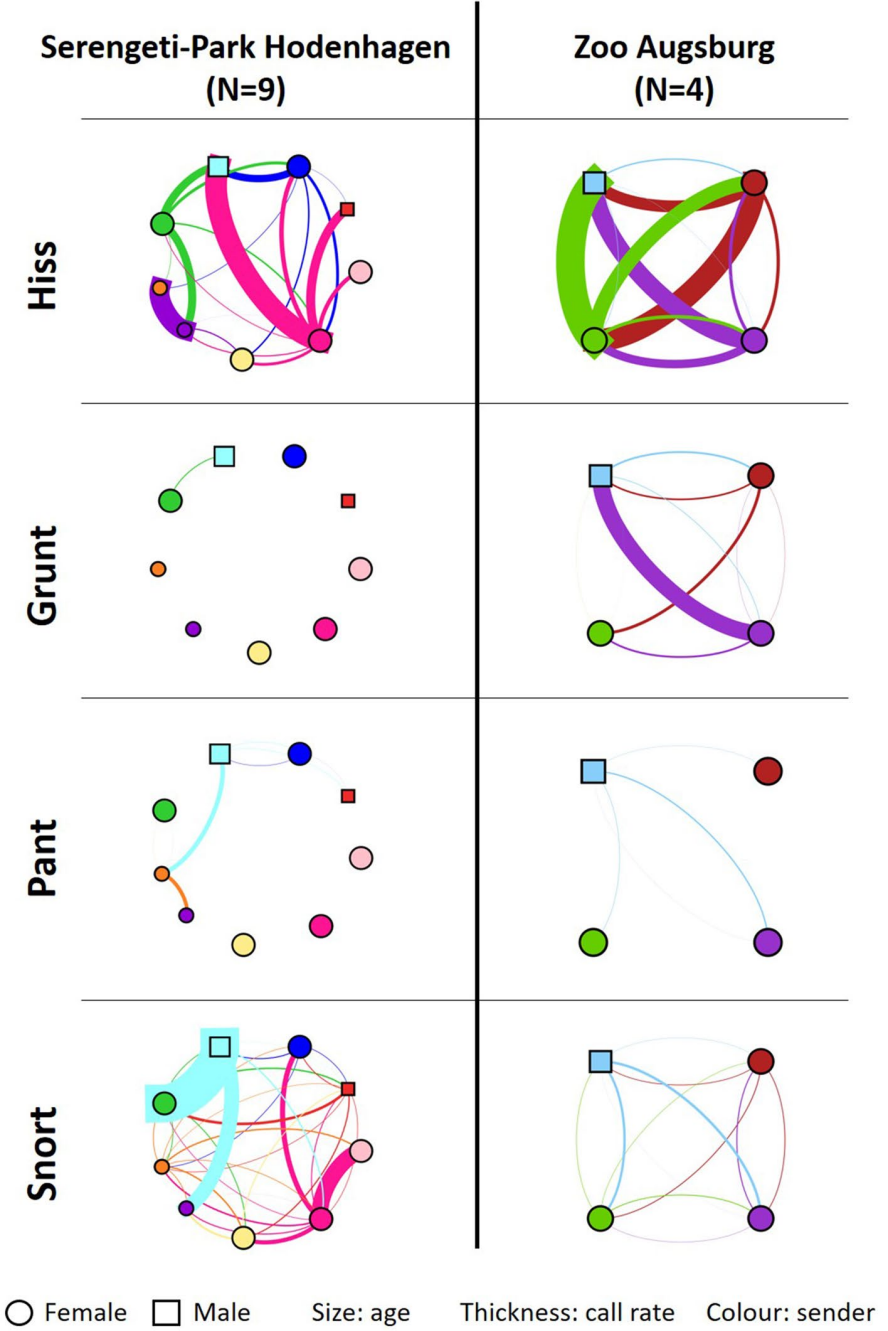
Exemplary vocal networks of Hiss, Grunt, Pant and Snort in two groups, Serengeti-Park Hodenhagen (N = 9) and Zoo Augsburg (N = 4). Nodes represent individuals and ties the dyadic call rate. Size of nodes corresponds to the age, larger nodes indicating older individuals. Colour of ties corresponds to colour of sender. Thicker ties indicate higher dyadic call rates [calls/contact hour], range: 0.2 calls/contact hour–190 calls/contact hour.

For all call types, a distinct communication structure was exposed, as none of the call rates in the networks were distributed equally between the dyads but rather heterogeneously, with some individuals emitting calls at a high rate to some of the potential receivers while not calling to others at all (Fig. 2, Supplementary 1+2).

For Hiss, Grunt and Pants, the adult individuals’ node as well as weight outdegrees did not correlate with the respective indegrees, suggesting an asymmetrical call rate distribution within the groups (− 0.083 ≤ r ≤ 0.293, *p* > 0.05, N = 26). While there was no significant correlation between weight in- and outdegrees for Snort either (r =  − 0.125, *p* = 0.543, N = 26), it was the only call type with a positive correlation between node out- and indegrees (r = 0.680, *p* < 0.001, N = 26), indicating a symmetrical relation between the number of conspecifics the individuals called to and those they were directed by.

### Sex-specific sender differences

Significant differences in overall call rates between adult females (N_Females_ = 19) and males (N_Males_ = 7) were found in Hiss and Pant. While females showed higher overall call rates for Hiss than males (mean_Females_ ± SD = 6.454 ± 7.405 calls/hour; mean_Males_ ± SD = 0.800 ± 0.684 calls/hour, t =  − 3.333, *p* = 0.004), males showed higher overall Pant call rates than females (mean_Females_ ± SD = 0.180 ± 0.322 calls/hour; mean_Males_ ± SD = 1.237 ± 1.242 calls/hour, t = 4.276, *p* < 0.001). In Grunt, there was a strong trend indicating that similar as in Hiss, females emitted more Grunts than males (mean_Females_ ± SD = 0.397 ± 0.621 calls/hour; mean_Males_ ± SD = 0.061 ± 0.162 calls/hour, t =  − 2.068, *p* = 0.053). No significant difference in overall Snort rates could be found between the sexes (mean_Females_ ± SD = 4.002 ± 1.572 calls/hour; mean_Males_ ± SD = 5.456 ± 2.509 calls/hour, t = 1.82, *p* = 0.085, Supplementary 4).

**Figure 3:**
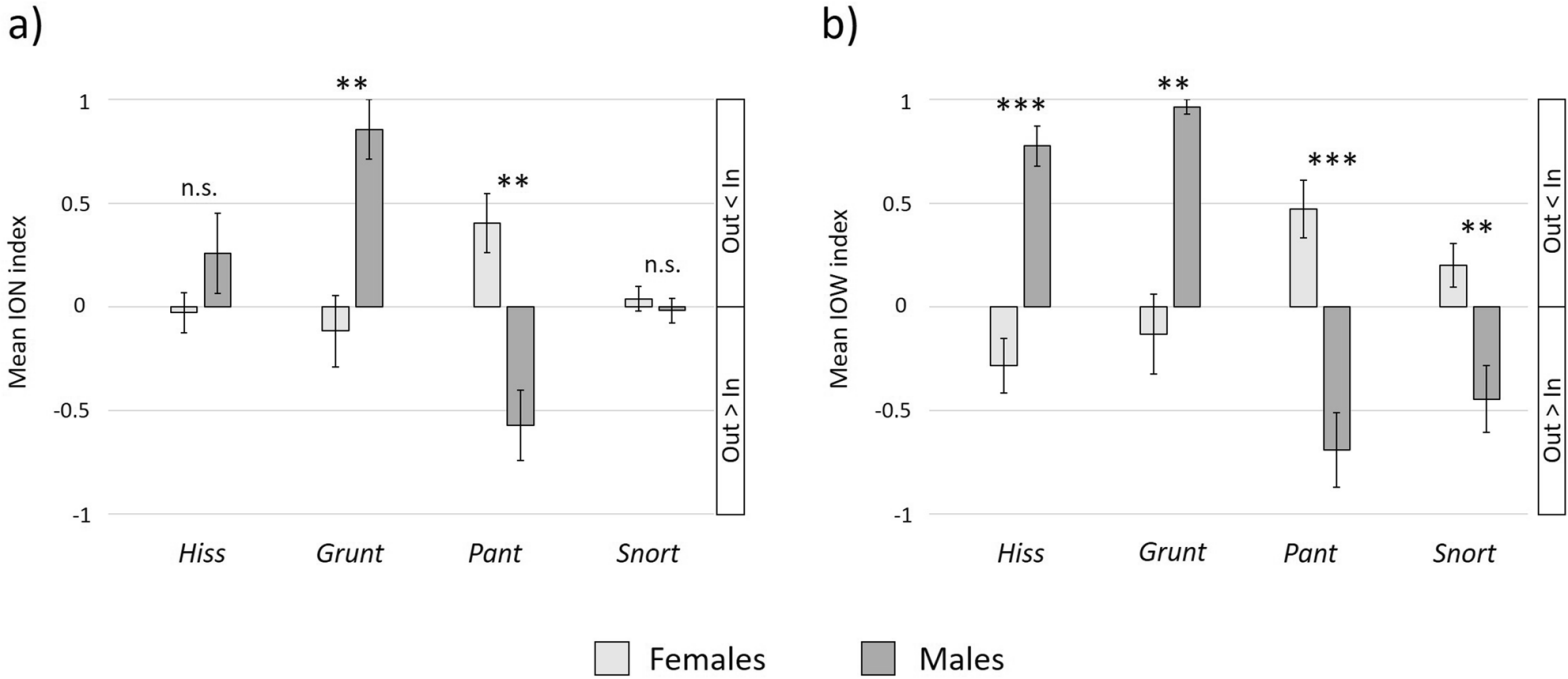
Mean and standard error of ION (**a**) and IOW (**b**) for female (N_Females_ = 19) and male (N_Males_ = 7) senders; **p ≤ 0.01, ***p ≤ 0.001; based on LMEs.

Significant differences in signalling distribution of incoming and outgoing node degrees (ION) between adult females (N_Females_ = 19) and males (N_Males_ = 7) were found for Pant (t =  − 3.579, *p* = 0.002) and Grunt (t = 2.942, *p* = 0.009), but not for Snort (t =  − 0.426, *p* = 0.675) and Hiss (t = 1.462, *p* = 0.161, Fig. 3a). Accordingly, males emitted Pant calls and females Grunt calls to more individuals than they received them from. Furthermore, there were significant differences in signalling distribution of incoming and outgoing weight degrees (IOW) found for each call type (Hiss: t = 4.576, *p* < 0.001; Grunt: t = 3.256, *p* = 0.004; Pant: t =  − 4.038, *p* < 0.001; Snort: t =  − 2.826, *p* = 0.011, Fig. 3b). More Snort and Pant calls were emitted by males rather than directed towards them, while females emitted more Hiss and Grunt calls than they received.

### Effects of dyad and interaction types

**Figure 4:**
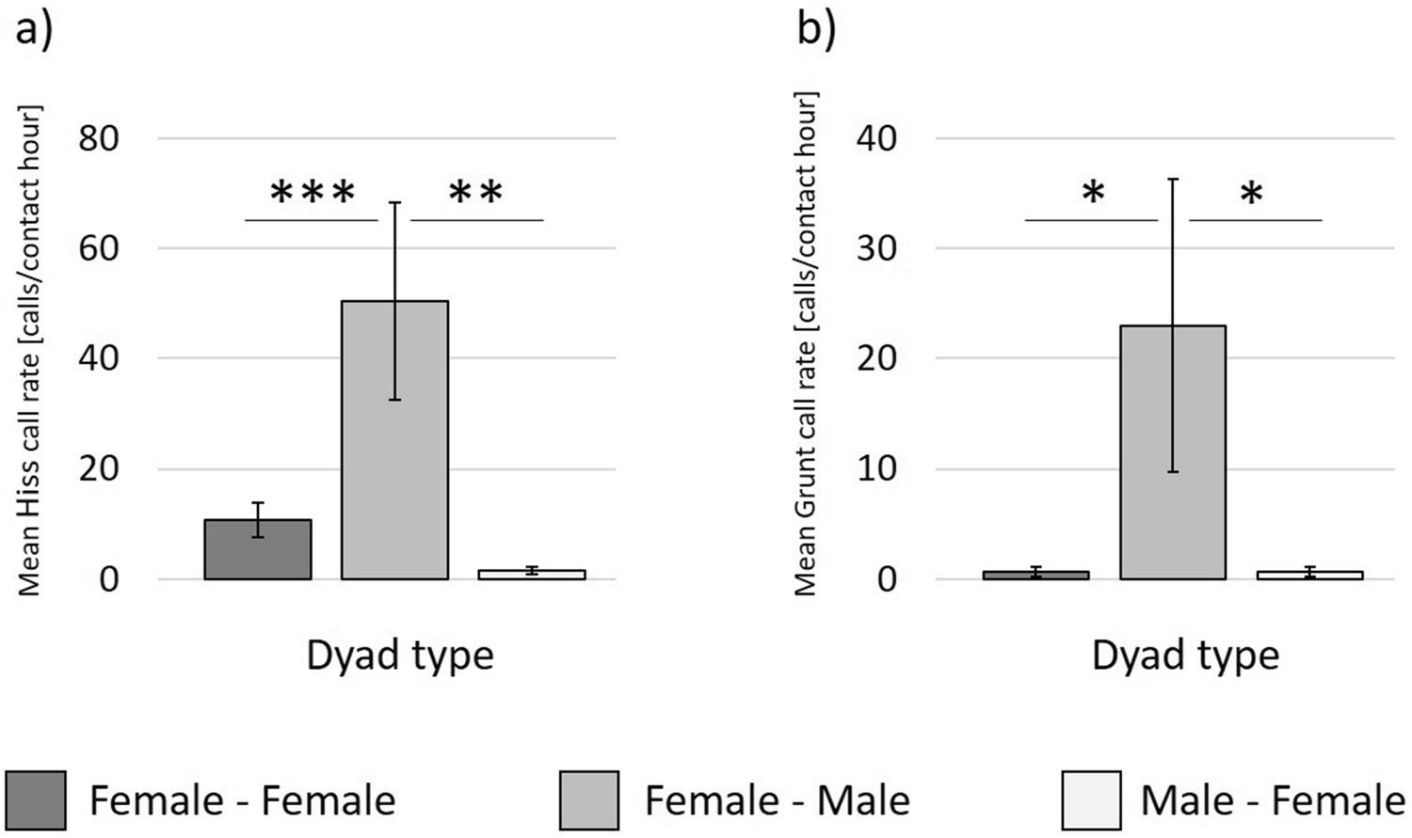
Mean and standard error of the dyadic Hiss call rate (**a**) and the dyadic Grunt call rate (**b**) for different dyad types; N_Female-Female_ = 40, N_Female-Male_ = 19, N_Male-Female_ = 19; first sex represents sender, second sex potential receiver. *p ≤ 0.05, **p ≤ 0.01, ***p ≤ 0.001; pairwise comparison based on Tukey adjustment.

For all call types, final models included only main factors but no interaction terms (Supplementary 3). For Hiss ‘dyad type’ turned out to have a significant effect on the dyadic call rate in all three social interaction rate models (*p* < 0.033). A subsequent comparison among dyad types revealed significantly higher call rates in female-male (N_Female-Male_ = 19) than in female-female (N_Female-Female_ = 40, t ≤ 4.608, *p* < 0.001) as well as in male–female (N_Male-Female_ = 19, t ≤ 3.378, *p* ≤ 0.020) dyads for affiliative and defensive interaction rate models (Fig. 4a). In the aggressive interaction rate model both main terms had a significant effect on Hiss call rate with call rates in female-male dyads (N_Female-Male_ = 19) showing a trend to be higher than in male–female (N_Male-Female_ = 19, t = 2.365, *p* = 0.072) but not in female-female (N_Female-Female_ = 40, t =  − 1.826, *p* = 0.172) dyads as well as increasing aggressive interaction rates predicting a rise in dyadic Hiss call rates (N_Dyads_ = 78, slope β = 8.718, SD =  ± 2.965, *p* = 0.005).

In Grunt ‘dyad type’ turned out to have similarly substantial effects as in the Hiss. In affiliative and defensive social interaction rate models, ‘dyad type’ significantly affected Grunt call rate (*p* ≤ 0.008), revealing significantly higher call rates in female-males dyads (N_Female-Male_ = 19) compared to female-female dyads (N_Female-Female_ = 40, t ≤ 2.778, *p* ≤ 0.022, Fig. 4b). The comparison between female-male (N_Female-Male_ = 19) and male–female (N_Male-Female_ = 19) dyads showed a significant difference in the affiliative interaction rate model (t = 2.628, *p* = 0.043) and a trend in the defensive interaction rate model (t = 2.315, *p* = 0.079). Unlike for Hiss, the effect of ‘dyad type’ on the dyadic Grunt call rate in the aggressive interaction rate model was only a trend (*p* = 0.077).

For Pant, a strong tendency towards ‘dyad type’ having an effect was found in the aggressive interaction rate model (*p* = 0.050), revealing that female-female dyads (N_Female-Female_ = 40) tended to emit lower Pant call rates than male–female dyads (N_Male-Female_ = 19, t =  − 2.323, *p* = 0.078). A further trend suggested that ‘defensive interaction rates’ had an effect on the dyadic call rate (*p* = 0.074), indicating higher Pant call rates in dyads with increased defensive interaction rates (N_Dyads_ = 78, slope β = 0.762, SD =  ± 0.417).

For Snort, the final models did not reveal any significant effects (*p* ≥ 0.118). Thus, neither the dyad nor the interaction types could sufficiently predict the differences for the dyadic Snort rate.

### Effects of dyad and association strength

For Hiss and Grunt the final model included an interaction between dyad type and association strength (Supplementary 3). The break down analysis revealed that only female-female dyads (N_Female-Female_ = 40) displayed significant negative coherences of association strength and dyadic call rate (Hiss: slope β =  − 64.652, SD =  ± 16.714, *p* = 0.001; Grunt: slope β =  − 9.566, SD =  ± 1.908, *p* < 0.001), suggesting that the stronger the associations between females, the lower the agonistic call rates between them.

There were no significant effects of association strength on the dyadic call rates of either Snort (*p* = 0.302) or Pant (*p* = 0.457), indicating that the level of association could not predict the respective call rates.

## Discussion

The findings of the present study prove that proximity-based communication structures provide a suitable approach for the analysis of vocal interactions in Southern white rhinoceros groups by revealing differences in call rates between female and male senders based on effects of the sex of potential receivers, the quality of social interactions as well as the association strength between group members.

Premised on the proximity-based approach, distinct vocal communication structures could be established for all four call types by displaying non-equal distributions of dyadic call rates within the groups. For three call types (Hiss, Grunt and Pant) an asymmetrical directionality was observed, which, according to Snijders and Naguib^4^ might provide information on the social relationship between group members. Indeed, significant differences in signalling distribution were found between sexes. Females, but not males, emitted higher rates of the agonistic call types Hiss and Grunt than they received, suggesting a more offensive social role of females compared to males. Conversely, the asymmetrical distribution of Pant call rates showed males, but not females, emitting more calls than they received, which suggests a rather active role of males in advertising behaviour. Snort*,* as the most frequent call type in the SWR, was the only call type showing a symmetrical call rate distribution. At the same time, there was no effect of sender or potential receiver and neither agonistic nor affiliative interactions could sufficiently predict the varying call rates. While the high occurrence and the symmetrical directionality might imply that Snorts are linked to cohesion behaviour, we could not find any relationship between the Snort call rate and association strength. Therefore, the social relevance as well as the communicational function of this call type remains to be clarified in further studies.

Differences between the signal transmission range of the call types and the set proximity measurement of one body length (2.5–3 m^46^) might have affected our findings, as calls uttered in long distance communication might be also detected from a greater distance than one body length and therefore, more individuals would need to be considered potential receivers. However, preceding pilot evaluations proved that none of the four call types was uttered more often in distant than close proximity. Quite on the contrary, Hiss and Grunt were uttered more frequently when conspecifics were at a close proximity rather than at a distant proximity, suggesting that the sender reacted to neighbours only when they were close enough to get in direct contact, posing an actual risk. Hence, one body length seems to reflect the distance that is most relevant for the occurrence of agonistic call types. Consequently, we would expect the distinct vocal network structure to dissolve and become more undifferentiated with increasing proximity measurement, as conspecifics that are out of reach for agonistic interactions would still be considered potential receivers. Pilot evaluations as well as previous studies showed that Pants and Snorts are emitted with the same probability in variable proximities to conspecifics^63^. Here, indeed, increasing the proximity measurement might affect the study’s outcome. However, while we would expect the dyadic call rates to turn out higher overall and, particularly for Pant, new sender-receiver dyads to appear in the communication networks, we do not expect the validity of the results to change significantly. We believe that the strongest dyads have already been detected by the initial proximity measurement (≤ 1 body length), as even though Pant calls might start at a distant proximity to neighbours, they continue during the locomotion of the sender while approaching others, therefore ending in close proximity. Moreover, both, Snorts and Pants, are uttered with closed mouths at a respectively low amplitude, which impedes the sound propagation and limits perception over great distances. Follow-up studies on signal transmission range of different call types in the SWR would help to determine the maximum distance at which conspecifics can still be considered potential receivers and therefore improve the definition of proximity measurements. Nonetheless, the distinct communication structures of all call types in the present study demonstrate that considering immediate neighbours as potential receivers is an applicable approach for constructing vocal networks when information on vocal exchange, bodily reactions or situational context is limited or entirely unavailable. Hence, call rates associated with the distance to a specific group member can be used in order to draw conclusions regarding the potentially intended receiver.

Regarding the call rates we were able to find sex-specific differences for the sender as well as an effect of the potential receivers, the quality of interaction between group members and the association strength, indicating a complex vocal communication structure in the SWR. Adult females emitted agonistic calls (Hiss and Grunt) at a higher rate and Pants at a significantly lower rate than males, which demonstrates that in SWR sex-specific differences in vocalisation are expressed in varying call rates, just as it has been shown in various other mammals as well (e.g.^14,25,30,34^). Furthermore, the significant effect of dyad type for both agonistic call types proved that not only the sex of the sender but also the sex of the potential receiver was decisive in the SWR vocal communication. Consequently, adult females did not only emit Hiss and Grunt at higher rates overall, but especially when males rather than other females were potential receivers. An effect of the receiver’s sex on the call rate has been found in other mammalian taxa as well (e.g.,^14,33^), suggesting that the constellation of the sex between sender and receiver is crucial for the call function. For example, in solitary living golden hamsters the highest call rates were emitted in opposite-sex dyadic encounters compared to same-sex encounters, suggesting the calls are elicited in a sexual context^33^. In contrast, in group-living non-human primates, females were observed to emit more affiliative calls to same-sex rather than to opposite-sex recipients, which is assumed to signal social preferences in female-bonded primate species^14^. A similar explanation was proposed for female African elephants that emitted more rumbles towards higher affiliated females^42^. Our present findings would therefore imply that adult female SWR predominantly use agonistic calls in opposite-sex contexts in order to keep the males at a distance. Even though there was a clear sex-specific difference in overall Pant rates between male and female senders, we could only find a trend in the effect of dyad type, indicating that female senders showed no preference for either sex of the potential receiver. Therefore, our results support the previous descriptions of Pant, as it appears to function as both, a socio-positive cohesive call type especially for the females^52,53,63,64^ as well as a mating call for the males^46^.

The quality of social interactions between sender and potential receiver predicted dyadic call rates for the Hiss call, allowing us to draw conclusions about the social role of group members. As dyads displaying high aggressive interaction rates showed respectively high Hiss call rates, it might be concluded that group members producing high Hiss rates are more aggressive and therefore more dominant than group members producing lower Hiss rates. This would fall in line with similar findings in other mammalian species where dominant group members exhibit higher call rates than subdominant ones (e.g.,^65–69^). Additionally, high defensive interaction rates showed a trend in predicting high Pant rates. The increase in Pant calls linked to a rise in defensive behaviour might be explained by males avoiding females’ aversive attitude when trying to approach and court them, which matches the fact that adult males uttered most of the Pant calls. Considering the differences in overall call rates for Hiss and Pant, the links between social interactions and dyadic call rates suggest that while female SWR play a dominant and often rejecting role especially towards adult males, male SWR appear more subordinate even though actively advertising at the same time. In light of these sex-specific behavioural and vocal characteristics, follow-up studies considering hormonal level and therefore accounting for the reproductive state of the animals would be of major interest, as the impact of hormonal state on the call rate has already been shown in several mammalian taxa^70–74^. These studies would complete the picture of factors that might affect the vocal communication in the SWR.

Last but not least, regarding the effect of association strength we could show that the closer two females were associated, the less they emitted agonistic calls (Hiss and Grunt) to each other, which might indicate social bonding between SWR females. Even though it might be argued that this is an effect of captivity^75^, temporarily stable female associations have also been reported in the field^51,76,77^. To date it needs to be clarified whether the decreased emission of agonistic calls between females is in fact indirect evidence of social preferences or if it is rather a reflection of social tolerance to specific group members.

Conclusively, our results show that even in species with loose social associations and low responsiveness, multiple factors influence the dynamics of their vocal communication network, allowing conclusions to be drawn concerning social relationships between group members. We demonstrated that by using proximity measurements, distinct vocal communication structures could be established for four of the most common call types in the captive SWR. Based on these structures we revealed sex-specific differences in call rates, especially for aggressive call types, with cows hissing and grunting more often especially at bulls, while bulls generally emit higher Pant rates. These findings fall in line with the white rhinoceros’ social organisation in the wild, therefore enabling further opportunities for applying the proximity-based approach for evaluating changes in group compositions and social associations. Overall, the study contributes to a deeper understanding of the link between vocalisation and behaviour as well as the possibility of social dynamics being reflected in vocal networks.

## Supplementary information

Supplementary Information.

## Data Availability

Raw data used for the manuscript are included in the supplementary information. Video and audio data are stored at the Institute of Zoology and are available on reasonable request.
