# Supplementary material for: Proximity-based vocal networks reveal social relationships in the Southern white rhinoceros
Source: Sci Rep. 2020 Sep 15;10:15104. doi: 10.1038/s41598-020-72052-0 (PMC7492360; doi:10.1038/s41598-020-72052-0)
Supplement: Supplementary file 1 — Supplementary Information. [file 41598_2020_72052_MOESM1_ESM.pdf]

Proximity-based vocal networks reveal social relationships in the Southern white rhinoceros

Julia Jenikejew[1]*, Brenda Chaignon[2], Sabrina Linn[3], Marina Scheumann[1]

[1]Institute of Zoology, University of Veterinary Medicine Hannover, 30559 Hannover, Germany

[2]Université Bourgogne Franche-Comté, 21078 Dijon, France

[3]Zoo Frankfurt, 60316 Frankfurt, Germany

*Corresponding author: Julia Jenikejew
University of Veterinary Medicine Hanover
Institute of Zoology
Bünteweg 17
30559 Hannover
Germany
julia.jenikejew@tiho-hannover.de

Supplementary Information

Supplementary 1: Data of vocal communication networks of Hiss, Grunt, Pant and Snort. ION = index of incoming and outgoing node degrees, IOW= index of incoming and outgoing weight degrees, range: -1 - +1. Min= minimum outgoing dyadic call rate (calls/contact hour), Max= maximum outgoing dyadic call rate (calls/contact hour), Mean= mean outgoing dyadic call rate (calls/contact hour).

			Hiss					Grunt					Pant					Snort				
ID	Sex	Zoo	ION	IOW	Min	Max	Mean	ION	IOW	Min	Max	Mean	ION	IOW	Min	Max	Mean	ION	IOW	Min	Max	Mean
Floris	M	Osnabrück	0.00	0.39	0.62	1.23	0.84	1.00	1.00	0.00	0.00	0.00	-1.00	-1.00	0.13	0.77	0.47	0.00	-0.22	3.20	6.05	4.33
Lia	F	Osnabrück	0.00	0.03	0.79	1.17	0.94	1.00	1.00	0.00	0.00	0.00	1.00	1.00	0.00	0.00	0.00	0.00	0.25	2.77	3.16	3.02
Marsita	F	Osnabrück	0.00	0.04	0.41	1.41	0.79	-1.00	-1.00	0.21	0.31	0.26	1.00	1.00	0.00	0.00	0.00	0.00	-0.28	2.83	8.88	4.98
Amalie	F	Osnabrück	0.00	-0.42	1.16	3.16	2.04	1.00	1.00	0.00	0.00	0.00	1.00	1.00	0.00	0.00	0.00	0.00	0.27	2.12	2.46	2.31
Bantu	M	Augsburg	0.00	0.93	1.07	6.70	3.35	0.00	0.75	0.23	8.71	3.89	-0.50	-0.95	1.10	5.85	2.93	0.00	-0.62	1.37	11.73	7.92
Baby	F	Augsburg	0.00	-0.40	14.76	100.83	68.35	-0.20	-0.40	0.94	13.51	7.41	1.00	1.00	0.00	0.00	0.00	0.00	-0.14	2.93	3.71	3.34
Kibibi	F	Augsburg	0.00	-0.62	14.31	81.06	44.49	-0.20	-0.92	0.78	74.55	28.03	0.00	0.93	0.00	0.21	0.07	0.00	0.51	0.21	4.55	1.98
Chris	F	Augsburg	0.00	-0.20	15.97	126.50	69.89	0.50	0.97	0.00	0.30	0.10	1.00	1.00	0.00	0.00	0.00	0.00	0.35	1.70	3.38	2.49
Dino	M	Erfurt	1.00	1.00	0.00	0.00	0.00	1.00	1.00	0.00	0.00	0.00	0.00	0.00	0.00	0.00	0.00	0.00	-0.65	3.39	14.22	8.80
Numbi	F	Erfurt	-0.33	-0.48	2.36	10.24	6.30	1.00	1.00	0.00	0.00	0.00	0.00	0.00	0.00	0.00	0.00	0.00	-0.03	3.03	3.79	3.41
Temba	F	Erfurt	-0.33	-0.87	4.41	29.85	17.13	-1.00	-1.00	0.44	4.01	2.22	0.00	0.00	0.00	0.00	0.00	0.00	0.66	0.70	3.03	1.86
Martin	M	Hodenhagen	1.00	1.00	0.00	0.00	0.00	1.00	1.00	0.00	0.00	0.00	0.00	-0.06	0.00	1.78	0.36	-0.33	-0.92	0.00	189.34	39.29
Molly	F	Hodenhagen	1.00	1.00	0.00	0.00	0.00	0.00	0.00	0.00	0.00	0.00	0.00	0.00	0.00	0.00	0.00	1.00	1.00	0.00	0.00	0.00
Claudia	F	Hodenhagen	-0.20	-0.21	0.00	36.12	11.41	0.00	0.00	0.00	0.00	0.00	0.00	0.06	0.00	1.59	0.32	0.00	0.70	0.00	4.39	0.88
Kianga	F	Hodenhagen	-0.50	-0.89	0.00	38.70	12.33	-1.00	-1.00	0.00	4.77	0.60	0.00	0.00	0.00	0.00	0.00	0.00	0.92	0.00	4.45	1.69
Uzuri	F	Hodenhagen	-0.43	-0.77	3.74	106.93	33.44	0.00	0.00	0.00	0.00	0.00	0.00	0.00	0.00	0.00	0.00	-0.50	-0.90	0.00	81.67	26.12
Lara	F	Hodenhagen	1.00	1.00	0.00	0.00	0.00	0.00	0.00	0.00	0.00	0.00	0.00	0.00	0.00	0.00	0.00	1.00	1.00	0.00	0.00	0.00
Lekuru	M	Gelsenkirchen	0.00	0.74	0.13	1.16	0.65	1.00	1.00	0.00	0.00	0.00	0.00	0.00	0.00	0.00	0.00	0.00	-0.02	3.81	3.85	3.83
Tamu	F	Gelsenkirchen	-0.33	-0.97	2.55	5.77	4.16	-1.00	-1.00	0.12	0.32	0.22	1.00	1.00	0.00	0.00	0.00	0.00	0.01	3.32	3.70	3.51
Cera	F	Gelsenkirchen	0.33	0.10	0.00	3.06	1.53	1.00	1.00	0.00	0.00	0.00	-1.00	-1.00	0.00	0.16	0.08	0.00	0.02	3.28	3.67	3.47
Kimba	M	Schwerin	0.00	0.32	0.73	1.06	0.89	1.00	1.00	0.00	0.00	0.00	-1.00	-1.00	0.29	0.31	0.30	0.00	0.20	2.32	2.83	2.58
Karen	F	Schwerin	0.00	-0.18	0.91	1.70	1.30	-1.00	-1.00	0.24	0.45	0.35	1.00	1.00	0.00	0.00	0.00	0.00	0.10	2.31	3.48	2.89
Clara	F	Schwerin	0.00	-0.18	1.08	1.74	1.41	1.00	1.00	0.00	0.00	0.00	1.00	1.00	0.00	0.00	0.00	0.00	-0.23	4.74	5.40	5.07
Harry	M	Münster	0.00	0.94	7.27	9.30	8.29	1.00	1.00	0.00	0.00	0.00	-1.00	-1.00	0.00	33.05	16.52	0.33	-0.88	0.00	25.24	12.62
Vicky	F	Münster	0.00	-0.15	20.21	174.47	97.34	-1.00	-1.00	0.00	121.73	60.87	0.00	0.00	0.00	0.00	0.00	-0.33	-0.81	1.15	4.80	2.98
Jane	F	Münster	0.00	-0.89	135.75	343.49	239.62	-1.00	-1.00	0.00	222.95	111.48	1.00	1.00	0.00	0.00	0.00	0.00	0.93	0.50	0.62	0.56

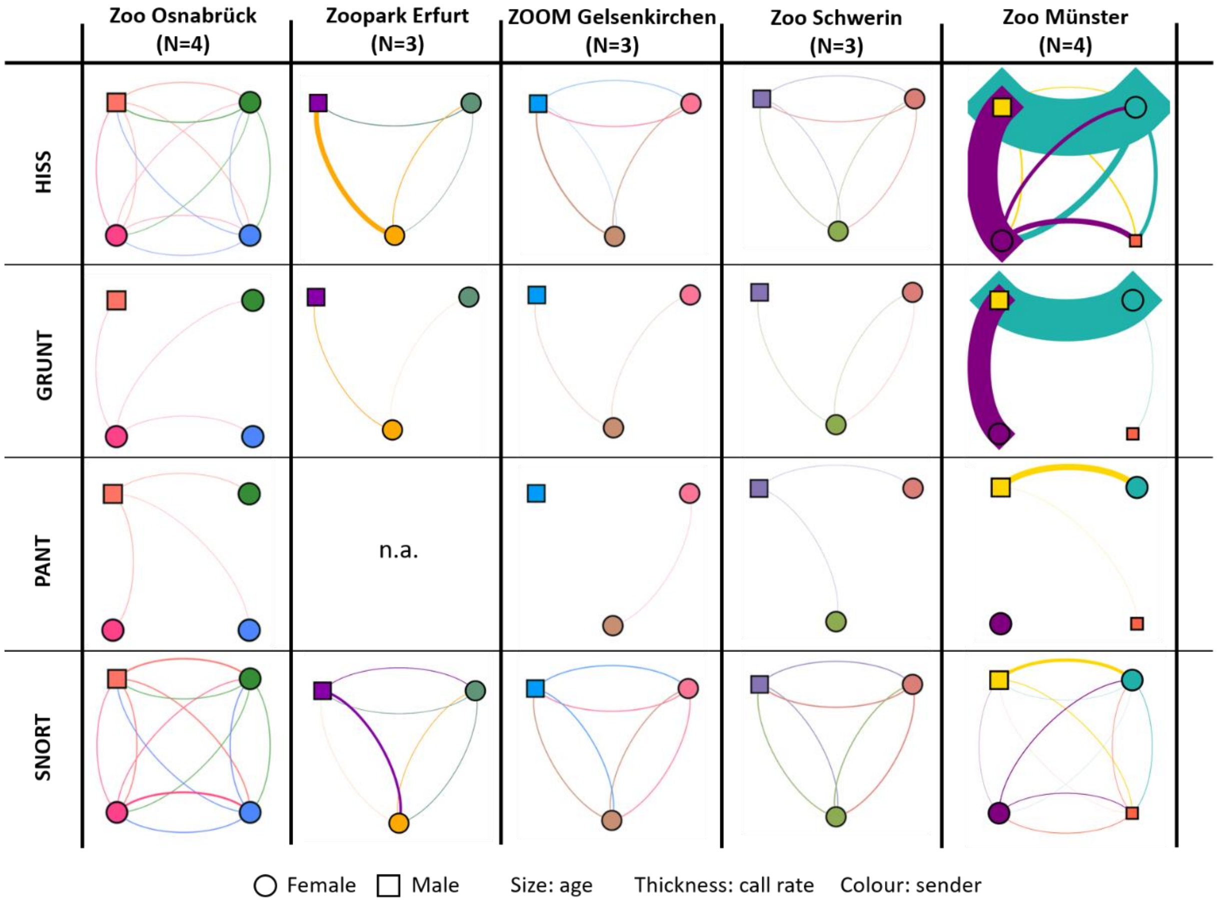

Supplementary 2: Vocal networks of Hiss, Grunt, Pant and Snort in five groups. Nodes represent individuals and ties the dyadic call rates. Size of nodes corresponds to the age, larger nodes indicating older individuals. Colour of ties corresponds to colour of sender. Thicker ties indicate higher dyadic call rates [calls/contact hour]; range: 0.1 calls/contact hour – 300 calls/contact hour.

Supplementary 3*:* Overview of results of Linear Mixed Effects (LME) models. Dyad=dyad type, F=female, M=male; first sex represents sender, second sex represents potential receiver. AS=association strength, Aff=affiliative interaction rate, Agg=aggressive interaction rate, Def=defensive interaction rate. Only final models are reported along with slope β values and standard deviation (±SD). The significance level was set at p ≤ 0.05, p < 0.1 was considered a statistical trend (T).

<table>
<tr><th></th><th colspan="3">Dyad type*Association Strength</th><th colspan="3">Dyad type*Affiliative interaction rate</th><th colspan="3">Dyad type*Aggressive interaction rate</th><th colspan="3">Dyad type*Defensive interaction rate</th></tr>
<tr><td rowspan="4">HISS</td><td colspan="3">Dyad*AS: p=0.002</td><td colspan="3">Dyad: p<0.001
Aff: n.s.</td><td colspan="3">Dyad: p=0.033
Agg: p=0.005, slope β=8.718, SD=±2.965</td><td colspan="3">Dyad: p<0.001
Def: n.s.</td></tr>
<tr><td colspan="3">break down analysis</td><td colspan="3">multiple comparisons</td><td colspan="3">multiple comparisons</td><td colspan="3">multiple comparisons</td></tr>
<tr><td>F-F</td><td>F-M</td><td>M-F</td><td>F-F vs F-M</td><td>F-F vs M-F</td><td>F-M vs M-F</td><td>F-F vs F-M</td><td>F-F vs M-F</td><td>F-M vs M-F</td><td>F-F vs F-M</td><td>F-F vs M-F</td><td>F-M vs M-F</td></tr>
<tr><td>p=0.001,
slope β=-64.652,
SD=±16.714</td><td>n.s.</td><td>n.s.</td><td>p<0.001</td><td>n.s.</td><td>p<0.01</td><td>n.s.</td><td>n.s.</td><td>p=0.04</td><td>p<0.001</td><td>n.s.</td><td>p<0.02</td></tr>
<tr><td rowspan="4">GRUNT</td><td colspan="3">Dyad*AI: p=0.021</td><td colspan="3">Dyad: p=0.008
Aff: n.s.</td><td colspan="3">Dyad: p=0.077 (T)
Agg: n.s.</td><td colspan="3">Dyad: p=0.008
Def: n.s.</td></tr>
<tr><td colspan="3">break down analysis</td><td colspan="3">multiple comparisons</td><td colspan="3">multiple comparisons</td><td colspan="3">multiple comparisons</td></tr>
<tr><td>F-F</td><td>F-M</td><td>M-F</td><td>F-F vs F-M</td><td>F-F vs M-F</td><td>F-M vs M-F</td><td>F-F vs F-M</td><td>F-F vs M-F</td><td>F-M vs M-F</td><td>F-F vs F-M</td><td>F-F vs M-F</td><td>F-M vs M-F</td></tr>
<tr><td>p<0.001,
slope β=-9.567,
SD=±1.908</td><td>n.s.</td><td>n.s.</td><td>p=0.022</td><td>n.s.</td><td>p=0.043</td><td>n.s.</td><td>n.s.</td><td>n.s.</td><td>p=0.021</td><td>n.s.</td><td>p=0.079 (T)</td></tr>
<tr><td rowspan="4">PANT</td><td colspan="3">Dyad: p=0.056 (T)
AI: n.s.</td><td colspan="3" rowspan="4">n.s.</td><td colspan="3">Dyad: p=0.050(T)
Agg: n.s.</td><td colspan="3" rowspan="4">Dyad: n.s.
Def: p=0.074 (T), slope β=0.762, SD=±0.417</td></tr>
<tr><td colspan="3">multiple comparisons</td><td colspan="3">multiple comparisons</td></tr>
<tr><td>F-F vs F-M</td><td>F-F vs M-F</td><td>F-M vs M-F</td><td>F-F vs F-M</td><td>F-F vs M-F</td><td>F-M vs M-F</td></tr>
<tr><td>n.s.</td><td>n.s.</td><td>n.s.</td><td>n.s.</td><td>p=0.078 (T)</td><td>n.s.</td></tr>
<tr><td>SNORT</td><td colspan="3">n.s.</td><td colspan="3">n.s.</td><td colspan="3">n.s.</td><td colspan="3">n.s.</td></tr>
</table>

Supplementary 4: Overall call rates (calls/observation hour). M=male, F=female.

Sender	SexSender	ZooID	HissRate	GruntRate	PantRate	SnortRate
Floris	M	Osnabrück	0.72	0.00	0.41	4.10
Lia	F	Osnabrück	0.73	0.00	0.00	3.13
Marsita	F	Osnabrück	0.41	0.20	0.00	2.53
Amalie	F	Osnabrück	1.56	0.00	0.00	2.29
Bantu	M	Augsburg	1.93	0.43	2.57	9.65
Baby	F	Augsburg	21.57	1.19	0.00	4.55
Kibibi	F	Augsburg	16.98	1.41	0.22	4.24
Chris	F	Augsburg	15.85	0.22	0.22	6.25
Dino	M	Erfurt	0.74	0.00	0.37	5.76
Numbi	F	Erfurt	2.55	0.00	0.00	3.88
Temba	F	Erfurt	4.59	0.37	0.00	3.28
Martin	M	Hodenhagen	0.00	0.00	3.19	7.79
Molly	F	Hodenhagen	0.00	0.00	0.00	0.21
Claudia	F	Hodenhagen	2.25	0.00	0.94	3.56
Kianga	F	Hodenhagen	3.45	0.22	0.43	4.31
Uzuri	F	Hodenhagen	7.73	0.21	0.21	6.87
Lara	F	Hodenhagen	0.44	0.00	1.11	4.88
Lekuru	M	Gelsenkirchen	0.70	0.00	0.00	4.98
Tamu	F	Gelsenkirchen	4.95	0.20	0.00	3.43
Cera	F	Gelsenkirchen	2.11	0.00	0.10	3.83
Kimba	M	Schwerin	0.10	0.00	0.50	2.77
Karen	F	Schwerin	0.72	0.10	0.20	4.40
Clara	F	Schwerin	1.11	0.00	0.00	4.66
Harry	M	Münster	1.42	0.00	1.62	3.14
Vicky	F	Münster	17.15	2.09	0.00	6.68
Jane	F	Münster	18.47	1.32	0.00	3.04
